# Research on Photoacoustic Synthetic Aperture Focusing Technology Imaging Method of Internal Defects in Cylindrical Components

**DOI:** 10.3390/s23156803

**Published:** 2023-07-30

**Authors:** Yanjie Zhang, Tianyou Li, Hongkai Chen, Zhihui Xu, Xinyao Li, Wangzhe Du, Yaxing Liu

**Affiliations:** 1 College of Mechanical and Vehicle Engineering, Taiyuan University of Technology, Taiyuan 030024, China; zhangyanjie@tyut.edu.cn (Y.Z.);; 2 Engineering Research Center of Advanced Metal Composites Forming Technology and Equipment, Ministry of Education, Taiyuan University of Technology, Taiyuan 030024, China; 3 National Key Laboratory of Metal Forming Technology and Heavy Equipment, Taiyuan University of Technology, Taiyuan 030024, China; 4 College of Architecture, Taiyuan University of Technology, Taiyuan 030024, China

**Keywords:** laser ultrasonic, synthetic aperture focusing technology, cylindrical components, two-wave mixing

## Abstract

Cylindrical components are parts with curved surfaces, and their high-precision defect testing is of great significance to industrial production. This paper proposes a noncontact internal defect imaging method for cylindrical components, and an automatic photoacoustic testing platform is built. A synthetic aperture focusing technology in the polar coordinate system based on laser ultrasonic (LU-pSAFT) is established, and the relationship between the imaging quality and position of discrete points is analyzed. In order to verify the validity of this method, small holes of Φ0.5 mm in the aluminum alloy rod are tested. During the imaging process, since a variety of waveforms can be excited by the pulsed laser synchronously, the masked longitudinal waves reflected by small holes need to be filtered and windowed to achieve high-quality imaging. In addition, the influence of ultrasonic beam angle and signal array spacing on imaging quality is analyzed. The results show that the method can accurately present the outline of the small hole, the circumferential resolution of the small hole is less than 1° and the dimensional accuracy and position error are less than 0.1 mm.

## 1. Introduction

Cylindrical components are widely used in various fields of industrial production, and the quality of cylindrical components directly determines the operating performance and service life of related equipment [1,2]. The internal defects of cylindrical components are prone to stress concentration under load, resulting in the expansion of defects and equipment damage [3,4,5]. Therefore, high-precision non-destructive testing methods for internal defects of cylindrical components are very important [6,7].

Among the commonly used non-destructive testing methods, penetrant testing (PT) and magnetic particle testing (MT) can only be used for surface crack detection [8,9]. Eddy current testing (ET) uses the principle of electromagnetic induction [10]. When the probe encounters a defect, it will cause a change in the impedance of the coil. By measuring the change of the electrical signal, it can be identified whether there is a defect, but ET can only detect surface or subsurface defects [11]. Radiographic testing (RT) uses the attenuation of rays to detect defects during the process of penetrating the sample. Since the absorption coefficient of defects on rays is different from that of the material itself, the shape and size of defects can be determined by the exposure rate of the film. However, rays are harmful to the human body, and the maintenance cost is high [12]. Ultrasonic testing (UT) uses the reflection of ultrasonic waves at defects to test samples. UT has the characteristics of high sensitivity, fast detection speed and good penetrating ability, and its signal can be processed in real time to determine the location and size of the defect. It has been gradually applied to the automatic detection of defects [13]. Common ultrasonic testing uses piezoelectric transducers as probes to test samples by contact or immersion. The signal is stable and the signal-to-noise ratio of this probe is high. It can perform high-precision detection of samples in the form of single-probe scanning or multi-probe phased array, and is a reliable contact detection method [14]. Single-probe scanning can draw B-scan or C-scan images through A-scan signals. B-scan images have no dynamic focus, and the accuracy of their defect detection is low, while C-scan images have high resolution when using high-frequency focusing probes. The multi-probe phased array integrates small piezoelectric chips into a probe, and realizes dynamic or virtual focusing through timing control or post-processing algorithms, with good imaging accuracy [15]. Laser ultrasonic (LU) is a non-contact ultrasonic testing method, which has the characteristics of no coupling agent and high temporal and spatial resolution [16,17,18]. LU can detect various defects of the sample without being limited by the shape of the part. For cylindrical components, real-time detection of defects can be realized by using its self-rotation.

Cylindrical components can be scanned and imaged using ultrasound signals. Ultrasonic B-scanning forms a two-dimensional data set from the echo signal obtained by the probe, and then uses the color scale to represent the numerical value of each array point in the data set to form a two-dimensional image [19]. Although B-scanning can achieve cross-sectional imaging, its resolution is low and it is difficult to accurately characterize defects [20]. Ultrasonic C-scanning uses the probe to scan along the surface of the sample, and can obtain images of the sample in the cross-section at a selected depth, but the size of the defect itself is difficult to determine [21]. Synthetic aperture focusing technology (SAFT) uses small aperture arrays to dynamically synthesize equivalent virtual large apertures, which can achieve high imaging resolution with small aperture arrays, and is especially suitable for high-resolution automatic detection of cylindrical components [22,23,24]. Dai [25] proposed an accurate frequency-domain synthetic aperture focusing technology based on 2D equivalent velocity mapping to detect and image internal defects in specimens. Ying [26] proposed a Multi-mode Time-domain SAFT algorithm, which can be used to superimpose SAFT images generated by single-mode ultrasound signals to obtain a better defect shape. Yoon [27] presented a method to enhance laser ultrasound image reconstruction with the time-domain synthetic aperture focusing technique, in which the acoustic velocity is extracted in situ with curve fitting. As for cylindrical components, Li [28] established a theoretical model of the forward vector algorithm for an ultrasonic immersion curved surface to improve the detection speed. Chen [29] proposed a new UT method using a hemispherical omnidirectional ultrasound probe and used SAFT to delay and beam-forming defect echoes to generate focused images of internal defects. Jin [30] proposed a frequency-domain SAFT for three-dimensional helical ultrasound imaging. The algorithm was established in the helical coordinate system to complete the synthetic aperture focusing process by adjusting the phase spectrum of the image. The above studies have proved the feasibility of UT for the detection of cylindrical components.

In this paper, laser ultrasonic is used to image the internal defects of cylindrical components, and a synthetic aperture focusing imaging algorithm in the polar coordinate system is established. The filtering method of the superimposed signal and the influence of parameters such as frequency range and array spacing on the imaging results are explored. The method proposed in this paper can accurately realize the imaging of internal defects of cylindrical components.

## 2. Experiments

### 2.1. Sample

**Figure 1:**
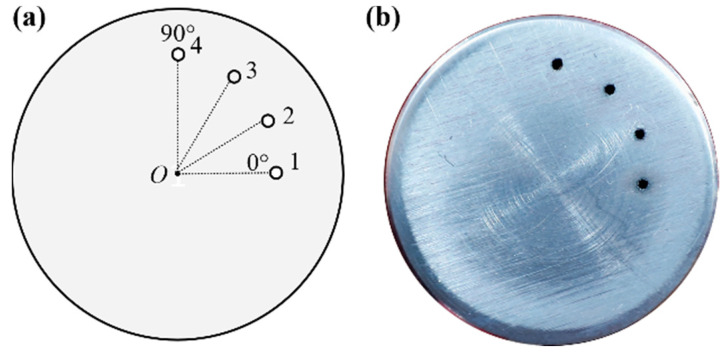
Sample tested in this experiment. (**a**) Schematic diagram of defects’ location; (**b**) the picture of the sample.

The sample used in this experiment was a 1060 aluminum alloy rod with a diameter of 20 mm, and its chemical composition is shown in Table 1. There are four holes with a diameter of 0.5 mm processed by an electric spark piercer on the side of the rod, as shown in Figure 1. The circumferential interval of the holes is 30°, and the radial interval is 0.5 mm, as shown in Table 2.

### 2.2. Laser Ultrasonic Imaging System

**Figure 2:**
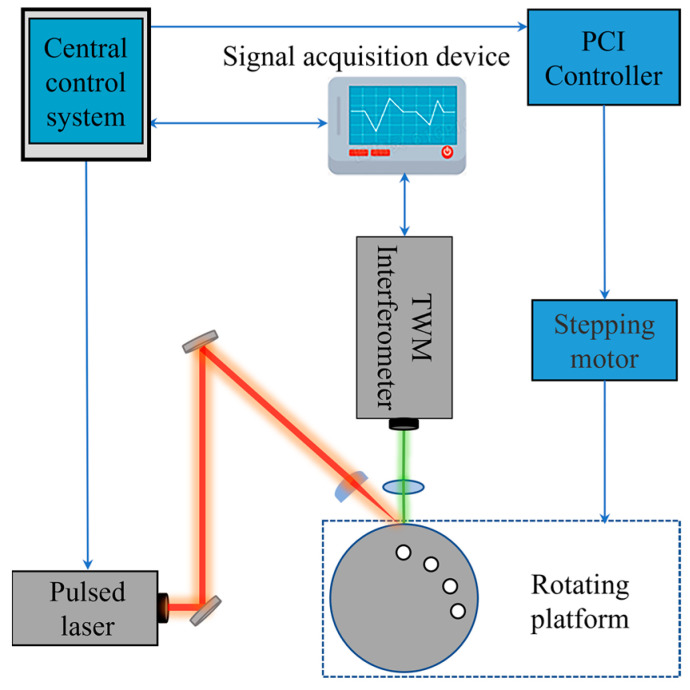
Schematic diagram of the experimental system.

The laser ultrasonic imaging system consists of a pulsed laser, two-wave mixing (TWM) interferometer, central control system, signal acquisition device and rotating platform, as shown in Figure 2.

Ultrasonic waves are excited by a pulsed laser, the wavelength of the pulse laser is 1064 nm, the repeat frequency is 10 Hz and the pulse width is 8 ns, which has a very high time resolution effect on the signal. The pulsed laser will leave a slight ablation on the surface of the sample after exciting the ultrasonic wave, which can be removed by gently grinding with sandpaper. Ultrasonic waves are detected by a TWM interferometer, and the two coherent beams in the photorefractive crystal are wavefront-matched, which ensures the TWM interferometer still has a good signal-to-noise ratio when measuring rough surfaces. In order to reduce the interference of the pulsed laser on the photodetector and the influence of local thermal expansion on the signal, the excitation beam and the detection beam are separated by 0.5 mm. During the signal acquisition process, the pulsed laser is focused on the surface of the aluminum alloy rod through a cylindrical lens into a line source with a width of 0.5 mm, and the ultrasonic waves are excited inside the rod. When the ultrasonic waves are reflected from the defects to the detection point, they are detected by the TWM interferometer and the signal is transmitted to the signal acquisition device, and the continuous wave laser used to detect ultrasonic waves can be focused into micron-sized spots with very high spatial resolution. The signal is single-shot-excited by a pulsed laser without averaging. When the signal of a detection point is collected, an electric pulse signal is sent to the stepper motor driver through the motion control card, and the stepper motor drives the rotating platform to make the sample turn to the next detection position with a step of 20′. The above steps are repeated until the scanning is completed.

**Figure 3:**
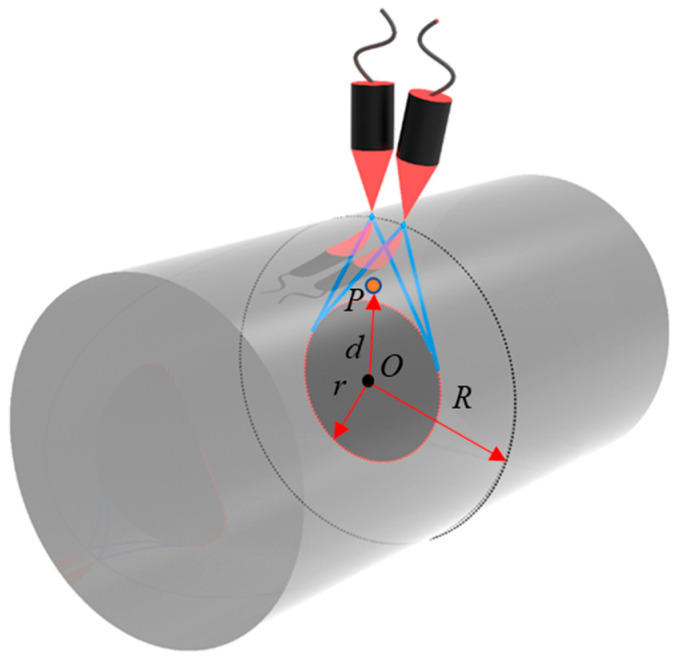
Cylindrical scanning diagram.

The scanning process is shown in Figure 3, where *P* is the point to be synthesized inside the rod, and *d* is the distance between *P* and the center of the cross section of the rod. During the scanning process, the overlapping part of the wave field excited by the probe in the rod will form an inscribed circle, and the radius *r* of the inscribed circle can be calculated as follows:

(1) $$r=R\sin\frac{\beta }{2}$$

where *β* is the ultrasonic beam angle, and *R* is the diameter of the rod. It can be seen from Figure 3 that the same point to be synthesized can be located in the wave field of the probe at different positions. Before imaging, it is necessary to first judge whether the imaging point is within the beam range, and then complete the signal synthesis through delay and superposition.

## 3. Methods

**Figure 4:**
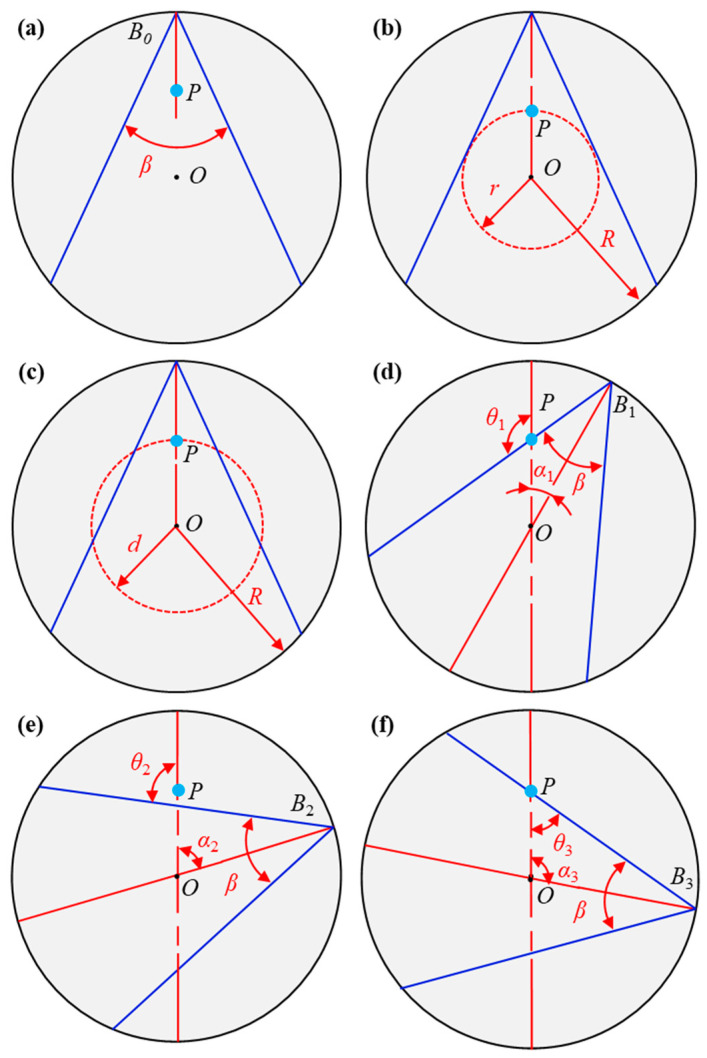
The scanning process of LU-pSAFT imaging. (**a**) Ultrasonic beam range at initial scan position *B*_0_; (**b**) intersection range of all beams, the inscribed circle with radius *r*; (**c**) trails of *P* beyond the inscribed circle of the ultrasonic beam; (**d**–**f**) the relationship between *P* and the ultrasonic beam range when the probe equivalently rotates clockwise.

The classical SAFT is mainly used for the inspection of the internal structure of samples with flat surfaces. In order to apply SAFT to the detection of defects in cylindrical components, an algorithm based on the polar coordinate system is proposed. In Figure 3, the radius of the rod is *R*, and the scanning step angle is Δ*α*. The process of the probe rotating around the rod is equivalent to the probe (excitation and detection lasers) being fixed and the rod rotating. Figure 4 is the schematic diagram of laser ultrasonic SAFT imaging scanning of the rod in the polar coordinate system (LU-pSAFT), where *B*_0_ represents the scanning starting point, *P* is the virtual focus point and the blue lines represent the beam range. When *B*_0_ scans around the rod, the intersection of all beam scanning ranges is an inscribed circle with *O* as the center and the beam boundary as the tangent, as shown by the red circle in Figure 4b; the radius of the inscribed circle is *r*. If the distance *d* from *P* to *O* is less than *r*, the trajectory of *P* is located in the inscribed circle, and the signals collected by all probes are involved in imaging. If the distance *d* from *P* to *O* is less than *r*, the trajectory of *P* is located in the inscribed circle, and the signals collected by all probes are involved in imaging. If *d* is greater than *r*, part of the trajectory of *P* will exceed the beam range, and some signals will not participate in the imaging of *P* during the scanning process, as shown in Figure 4c. It can be seen that the number of signals involved in imaging is related to the distance between *P* and *O*. The smaller *d* is, the deeper the radial depth of *P*, and the more signals are involved in the imaging process, which coincides with the classical SAFT algorithm.

Figure 4d–f show the ranges of the ultrasonic beam when the probe rotates clockwise. When the probe rotates from *B*_0_ to *B*_1_, *P* is always within the beam range; until the probe rotates to *B*_1_, *P* is located at the beam boundary, as shown in Figure 4d. When the probe rotates from *B*_0_ to *B*_1_, *P* is always within the beam range; until the probe rotates to *B*_1_, *P* is located at the beam boundary, as shown in Figure 4d. When the probe continues to rotate, e.g. at the position of *B*_2_, *P* is beyond the beam range, as shown in Figure 4; the ultrasonic signal at this position is not used for the imaging of *P*. Until the probe rotates to *B*_3_, *P* re-enters the beam range, as shown in Figure 4f.

It is clearly that when the probe scan from *B*_0_ to *B*_1_*, P* is within the wave field coverage; when the probe scan from *B*_1_ to *B*_2_, *P* exceeds the wave field coverage. Therefore, it is necessary to explore the angle of the probe that does not participate in the imaging of *P* when *P* exceeds the inscribed circle.

It is assumed that the ultrasonic beam excited in the rod by the pulsed laser at all positions is the same, namely that *β* is constant and that the pulsed laser is well aligned. In Δ*OB*_1_*P* and Δ*OB*_3_*P* in Figure 4d,f, according to the sine law:

(2) $$\{\begin{array}{l} d\sin\left(\theta_{1}\right)=R\sin\frac{\beta }{2}\\ d\sin\left(\theta_{3}\right)=R\sin\frac{\beta }{2}\\ \alpha_{1}=180{}^{\circ}-\theta_{1}-\frac{\beta }{2}\\ \alpha_{3}=180{}^{\circ}-\theta_{3}-\frac{\beta }{2} \end{array}$$

In Figure 4d, *θ*_1_ is an obtuse angle, and in Figure 4f, *θ*_3_ is an acute angle. *θ*_1_ and *θ*_3_ can be calculated as:

(3) $$\{\begin{array}{l} \theta_{1}=180{}^{\circ}-\arcsin\left(\frac{R}{d}\sin\frac{\beta }{2}\right)\\ \theta_{3}=\arcsin\left(\frac{R}{d}\sin\frac{\beta }{2}\right) \end{array}$$

Combining Equations (2) and (3), the rotation angles *α*_1_ and *α*_3_ can be calculated as follows:

(4) $$\{\begin{array}{l} \alpha_{1}=\arcsin\left(\frac{R}{d}\sin\frac{\beta }{2}\right)-\frac{\beta }{2}\\ \alpha_{3}=180{}^{\circ}-\arcsin\left(\frac{R}{d}\sin\frac{\beta }{2}\right)-\frac{\beta }{2} \end{array}$$

According to the symmetry of the rod, on the other side of the center line, there is also a probe range where the ultrasonic beam does not cover *P*. Therefore, during the scanning process, the probe range *γ* that cannot form effective imaging for the part beyond the inscribed circle can be expressed as:

(5) $$\{\begin{array}{l} \gamma_{1}=\alpha_{3}-\alpha_{1}\\ \gamma_{2}=\gamma_{1}\\ \gamma =\gamma_{1}+\gamma_{2} \end{array}$$

where *γ*_1_ and *γ*_2_ are the angle ranges of probes not participating in *P* imaging on the left and right sides of the center line, respectively. Combining Equations (4) and (5), *γ* can be expressed as:

(6) $$\gamma =360{}^{\circ}-4\arcsin\left(\frac{R}{d}\sin\frac{\beta }{2}\right)$$

It can be seen from Equation (6) that the position of the probe involved in imaging is related to the radial depth of the point to be synthesized, when *P* is closer to *O* (i.e., the deeper the radial depth), the greater the number of signals involved in imaging, the higher the image resolution. When *d* is smaller than *r*, all signals participate in the imaging of *P*, which is consistent with Figure 4b.

**Figure 5:**
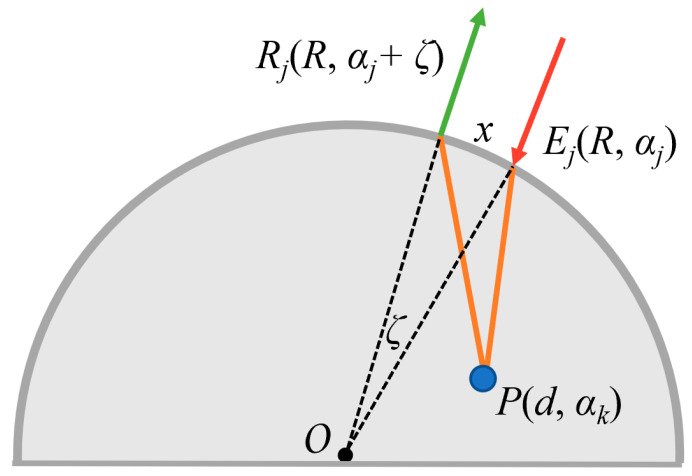
Propagating time calculation schematic.

Because of the different positions of the pulsed lasers, the propagation path of the ultrasonic waves passing through the virtual focal point is different, and the propagating time must be calculated in order to realize imaging. The calculation principle of the propagating time in the polar coordinate system is shown in Figure 5.

In Figure 5, the center of the rod is *O*, and the coordinates of *P* in the polar coordinate system are (*d*, *α_k_*). The coordinates of the excitation *E_j_* and detection *R_j_* are (*R*, *α_j_*) and (R, *α_j_* + *ζ*), respectively, and the arc length between *E_j_* and *R_j_* is *x* (approximately the length of a straight line). The central angle between *E_j_* and *R_j_* is ζ. Based on the principle of synthetic aperture focusing and the equation for the distance between two points in the polar coordinate system, the propagating time *t_kj_* of the signal received at *R_j_* can be calculated as:

(7) $$\{\begin{array}{c} x=\xi R\\ t_{kj}=\frac{\sqrt{R^{2}+d^{2}-2Rd\cos\left(\alpha_{j}-\alpha_{k}\right)}+\sqrt{R^{2}+d^{2}-2Rd\cos\left(\alpha_{j}+\xi -\alpha_{k}\right)}}{c} \end{array}$$

where *R* is the diameter of the rod and *c* is the velocity of the longitudinal wave. The signal intensity *S_SAFT_(t*) at *P* to be synthesized can be calculated by the superposition of signal amplitudes corresponding to the propagating time as follows:

(8) $$S_{SAFT}\left(t\right)=\frac{\sum_{1}^{n}S_{j}\left(t_{kj}\right)}{n}$$

where *n* represents the number of signals involved in imaging and *S_j_* is the signal received at *R_j_*.

The LU-pSAFT imaging process in this experiment can be described as follows:

1. The rod rotates one circle along the axis with the step angle Δα to obtain the original set of laser ultrasonic signals.
2. The rod section in the polar coordinate system is discretized to obtain the polar coordinate data matrix.
3. According to Equation (6) and the radial depth *d* of the discrete point, the range of the signal area participating in the imaging at this point is calculated. Then, imaging calculations are performed for each discrete point according to Equations (7) and (8).
4. The data matrix in the polar coordinate system is drawn in the form of a contour to realize LU-pSAFT imaging.

## 4. Analysis

### 4.1. Signal Analysis and Processing

**Figure 6:**
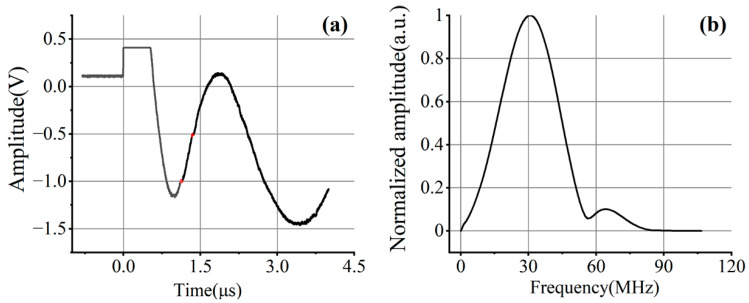
(**a**) Typical laser ultrasonic signal; (**b**) frequency spectrum of the longitudinal wave. Since the amplitude of the longitudinal wave reflected by the small hole is too weak, the same experimental parameters are used to obtain the longitudinal wave in a 1060 aluminum plate with a thickness of 5 mm.

A typical signal collected during the experiment is shown in Figure 6a. Longitudinal waves were used to image in this experiment. However, the amplitude of the longitudinal wave reflected by the hole was very small, so the spectrum of the longitudinal wave signal in another 1060 aluminum plate was extracted to determine the frequency range of the longitudinal wave, as shown in Figure 6b. The longitudinal wave was detected on the opposite side of the excitation point and aligned on the center. The longitudinal wave spectrum of the plate cannot replace that of a cylindrical sample, and can only be used as a reference to narrow the frequency range of filtering and verify the feasibility of filtering.

**Figure 7:**
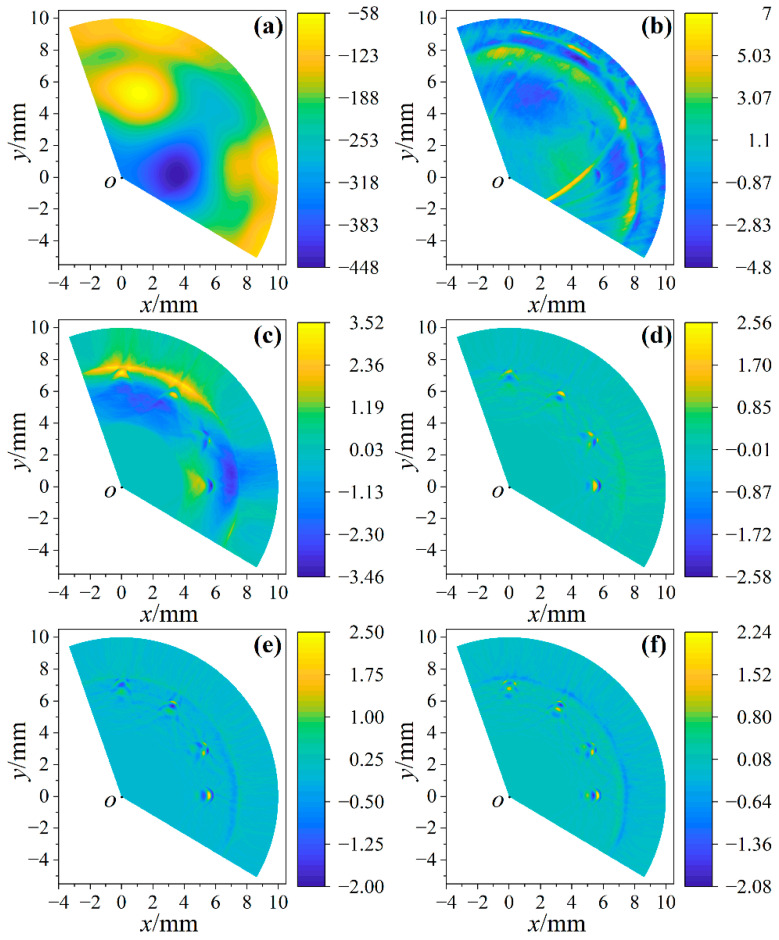
Imaging results after signal processing. (**a**) Imaging by original signals; (**b**) imaging by signals filtered once; (**c**–**f**) imaging by signals windowed after being filtered once, twice, thrice and four times.

As can be seen in Figure 6a, the pulsed laser excites a complex signal in the rod. After the signal is triggered, the photodetector is affected by the intense pulse laser and thermal expansion, and the amplitude of the signal exceeds the display range of the oscilloscope, so a horizontal line is displayed. After the amplitude falls back, the overall signal shows a waveform similar to a Lamb wave, and its amplitude is much larger than the longitudinal wave reflected by the small hole (the red signals in Figure 6a), while clutters cause serious interference to the imaging process. The red signals move during the scanning process, which is related to the reflection time of the longitudinal wave at the small hole. It can be seen from the spectrum that the longitudinal wave excited by the pulsed laser is broadband and has a center frequency around 30 MHz. Therefore, a Butterworth filter in the band-pass range of 5–55 MHz was selected for preliminary filtering, and the imaging results are shown in Figure 7.

Figure 7a is the imaging result based on the original signals. Since the amplitude of the clutter is much larger than the longitudinal wave used for imaging, it is difficult to show defects in the image. Figure 7b shows the imaging result after signals were filtered by a 5–55 MHz band-pass filter. In the imaging result, the outline of defects appears faintly, which is difficult to distinguish accurately. According to the laser ultrasonic signal in Figure 6a, it can be seen that the signal has a high amplitude when the signal is triggered, which has a great influence on the imaging quality near the rod surface, so signals need to be windowed to eliminate the amplitude of the triggering signal. Figure 7c is the imaging result after signals pass through a band-pass filter and are windowed. It can be seen that defects are slightly clearer due to the reduced amplitude near the surface. In order to further improve the image quality, signals were filtered multiple times. Figure 7d–f show the imaging results of signals after being filtered twice, thrice and four times, respectively (signals are windowed after being filtered). It can be seen that the image quality when signals are filtered twice and thrice is better than that when they are filtered four times, and artifacts appear in the image after signals are filtered four times, which shows that too many instances of filtering cannot improve the image quality.

**Figure 8:**
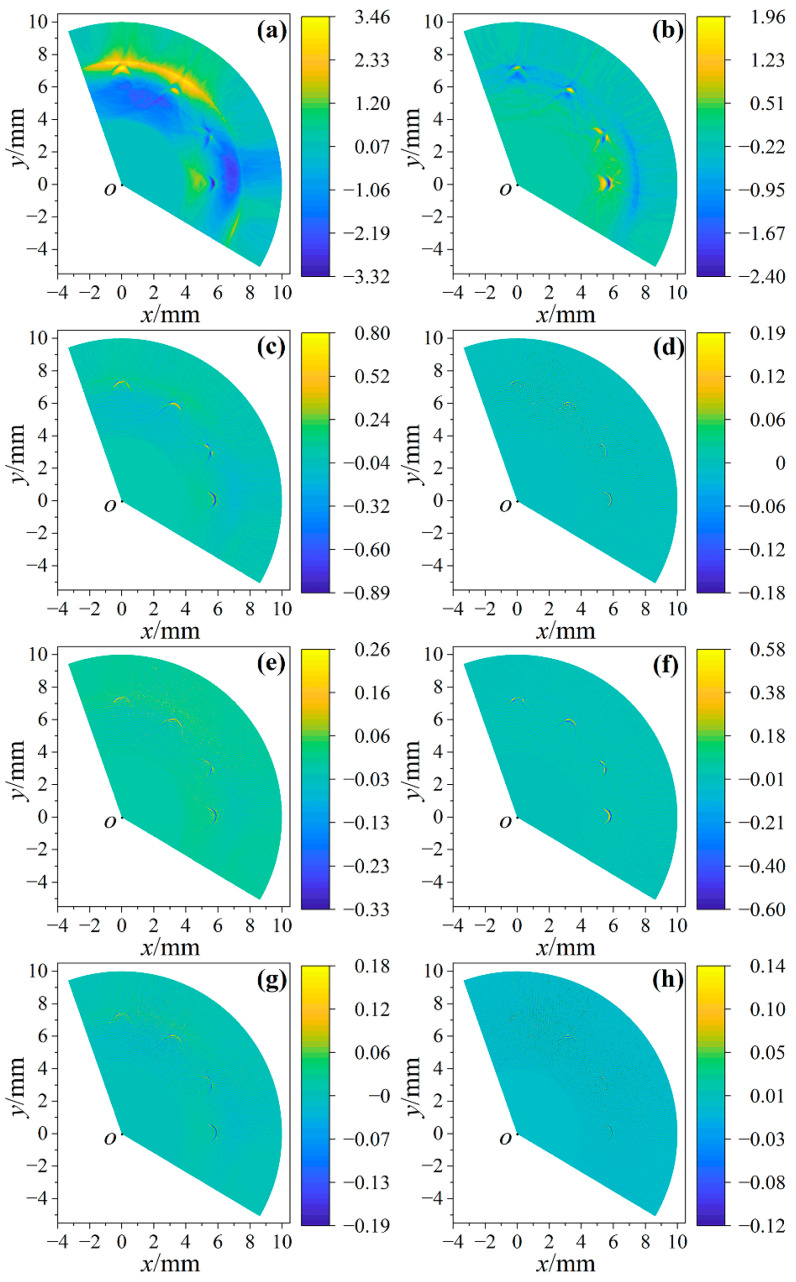
Imaging by signals windowed after being filtered twice and thrice. (**a**,**c**,**e**,**g**) are the imaging results of signals filtered twice by 5–15 MHz, 15–25 MHz, 25–35 MHz and 35–45 MHz band-pass filters; (**b**,**d**,**f**,**h**) are the imaging results of signals filtered thrice by the same band-pass filters as in (**a**,**c**,**e**,**g**).

**Figure 9:**
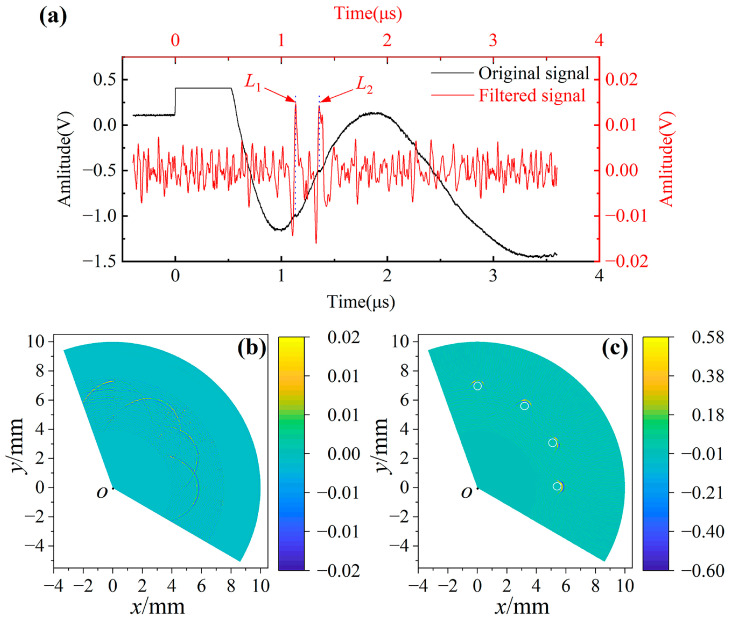
Comparison of B-scan with LU-pSAFT imaging results. (**a**) Comparison of original signal and filtered signal; (**b**) B-scan image; (**c**) LU-pSAFT imaging results, white circles are actual holes.

The broadband band-pass filter eliminates the influence of clutters on imaging, but it is difficult to present defects accurately due to the large frequency range. Therefore, it is necessary to narrow the frequency range of the band-pass filter to achieve clear imaging. Figure 8a–h show the imaging results of signals filtered twice and thrice when the frequency range of the band-pass filter is 5–15 MHz, 15–25 MHz, 25–35 MHz and 35–45 MHz, respectively. In order to reliably assess the quality of images, the signal-to-noise ratios (SNRs) of images were calculated. The ratio of the mean and standard deviation of all pixels was used to calculate the SNR of the image. The higher the absolute value of SNR, the better the image quality. In Figure 8, all the twice-filtered images and the thrice 5–15 MHz filtered images have obvious low-frequency shading, so the images filtered thrice at 5–15 MHz, 15–25 MHz and 25–35 MHz are compared. The SNRs of Figure 8d,f,h are −0.006, 0.016 and 0.006, respectively, so Figure 8f has the highest image quality, which indicates that the imaging quality of the signals filtered by the 25–35 MHz band-pass filter was the highest, and the arc of the defect can be clearly observed. One of the filtered signals used for imaging in Figure 8f is shown as the red line in Figure 9a. The black signal in Figure 9a passes through 25–35 MHz band-pass filters thrice to extract the reflected longitudinal wave of the small hole. *L*_1_ and *L*_2_ are longitudinal waves reflected by small holes, and their amplitude and position change dynamically during the scanning process.

**Figure 10:**
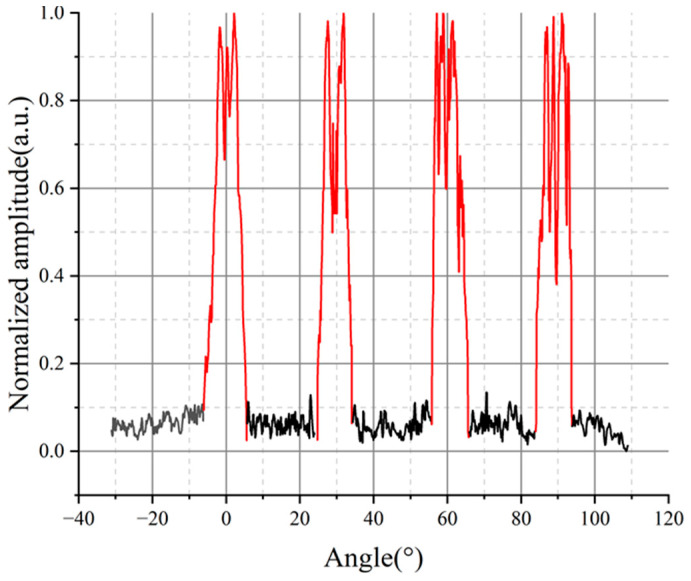
Circumferential resolution of holes.

In order to verify the effectiveness of the method proposed in this paper, it is compared with the traditional B-scan results. As shown in Figure 9b, the B-scan image shows the existence of defects inside the rod, but the effective information of the defects cannot be obtained from the imaging results, while the pSAFT algorithm used in this experiment can effectively present the outline of small defects with an accurate position. In Figure 9c, the pixel maxima at each angle are selected and normalized to obtain the circumferential resolution of holes. The red line in Figure 10 represents the measured position of holes, and the position errors are calculated using −6 dB. When calculating the range limits and sizes, the valid part of the hole’s outline is selected. The angle errors, position errors and size errors are shown in Table 3.

### 4.2. Effect of β on Image Quality

**Figure 11:**
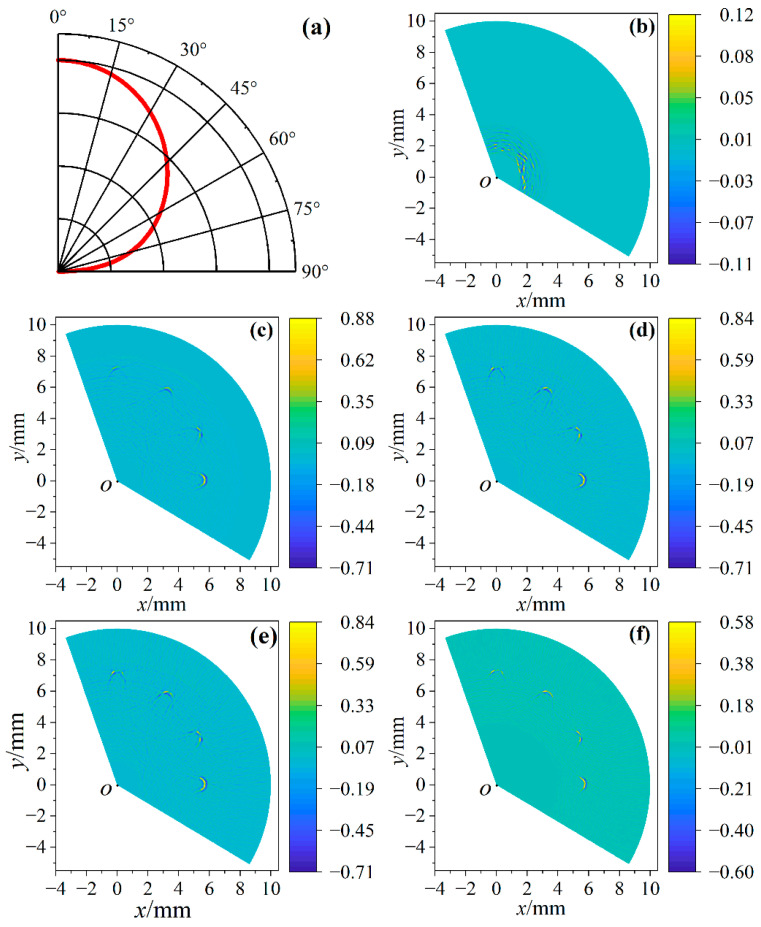
The relationship between *β* and image quality. (**a**) Directionality and intensity of laser-induced longitudinal wave. Due to symmetry, *β* is twice the directivity angle; (**b**–**f**) are the imaging results when *β* is 20°, 60°, 100°, 140° and 180°, respectively.

Figure 11a is the intensity and direction curve of the longitudinal wave excited by the pulsed laser. It can be seen that the intensity of the longitudinal wave is the strongest in the normal direction, and gradually weakens with the increase of the angle. It can also be seen from Equation (6) that the number of signals involved in imaging is related to *β*. As the angle increases, it is difficult for longitudinal waves to have effective echoes at small defects, and noise signals may even appear. Therefore, it is necessary to select *β* in the imaging process to achieve the best imaging quality.

Figure 11b shows the imaging result when *β* = 20°. Because the ultrasonic beam angle is too small, there are too few signals involved in imaging, and it is difficult to show defects. Figure 11c–f are the imaging results when *β* is 60°, 100°, 140° and 180°, respectively, and the SNRs of Figure 11c–f are −0.008, −0.018, −0.016 and −0.016, respectively. It can be seen that when *β* = 100°, the image quality is the best, and the insufficient number of signals participating in the imaging will lead to the loss of effective defect echo information; when the signals with a lower amplitude of defect echoes participate in the imaging, the signal-to-noise ratio of the imaging will decrease. Therefore, in this experiment, choosing an ultrasonic beam angle near 100° obtained the best imaging quality.

### 4.3. Effect of Laser Array Spacing on Imaging Quality

**Figure 12:**
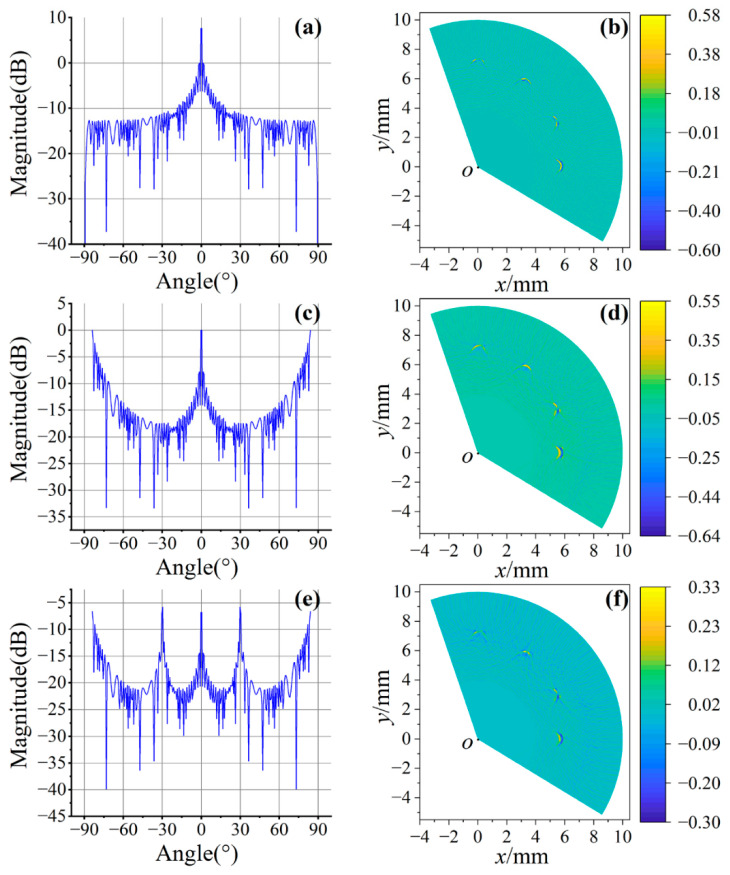
The effect of array spacing on the imaging quality. (**a**,**c**,**e**) are the results of *P*(*θ*) when *D* is *λ*/4, *λ*/2 and *λ*, respectively; (**b**,**d**,**f**) are imaging results when *D* is *λ*/4, *λ*/2 and *λ*, respectively.

In LU-pSAFT processing, in general, the higher the density of the signal array, the higher the image quality. However, acquiring laser ultrasonic signals requires scanning point by point, and too-small array spacing leads to a decrease in the efficiency of signal acquisition. Therefore, it is necessary to explore the optimal array spacing to achieve the optimum imaging efficiency. The beam *P*(*β*) of the virtual linear array can be expressed as:

(9) $$P\left(\beta \right)=K\frac{\sin\left(k_{0}ND\sin\beta \right)}{\sin\left(k_{0}D\sin\beta \right)}$$

where *k*_0_ is the wave number and *β* represents the beam angle, *D* is the array spacing of lasers and *N* represents the number of signals, and *D* and *N* are inversely proportional when the sweep range is certain. In this experiment, the velocity of the longitudinal wave was about 6300 m/s, and the center frequency was about 31 MHz, so the wavelength of the longitudinal wave was about 0.2 mm. The images for different *D* are shown in Figure 12.

Figure 12a,b show the beam and imaging results when *D* = *λ*/4. It can be seen that the width of the main lobe is narrow and the imaging quality is high (SNR = 0.016). When *D* = *λ*/2, a large side lobe appears at ±90°, as shown in Figure 12c, and some noise appears in the image of Figure 12d (SNR = 0.009). When *D* = *λ*, the beam in Figure 12e has multiple main lobes, and the imaging quality in Figure 12f is greatly reduced (SNR = 0.0003). It can be seen that a smaller array spacing will have better imaging quality, but the time consumed by signal acquisition should be considered. Although the array spacing can be further reduced, when *D* = *λ*/4, the defects are clearly discernible and the image noise is small, which can achieve the optimal imaging efficiency.

## 5. Conclusions

In this paper, a photoacoustic imaging method for internal defects of cylindrical components is proposed, and the algorithm is established in the polar coordinate system. Affected by the range of the ultrasonic beam, discrete points outside the inscribed circle of the ultrasonic beam cannot utilize all signals for imaging, so the range of signals used for imaging of these discrete points is calculated.

Holes with a diameter of 0.5 mm were tested in the experiment, and the signals were filtered multiple times and windowed before the weak longitudinal waves echoes could be extracted to images. The intensity of the laser-induced longitudinal wave decreased with the increase of the angle in the normal direction. If the beam angle used for imaging is too large, the signal-to-noise ratio of the image will decrease due to the decrease of the intensity of the longitudinal wave. In addition, the influence of signal array spacing on imaging quality is analyzed. Optimal imaging with the highest signal acquisition efficiency can be achieved when *D* = *λ*/4. The results show that this method can accurately present the outline of the small hole, the circumferential resolution of the small hole is less than 1° and the dimensional accuracy and position error are less than 0.1 mm.

## Figures and Tables

**Table 1:** Chemical composition of 1060 aluminum alloy.

Si (%)	Cu (%)	Mg (%)	Zn (%)	Ti (%)	Mn (%)
0.25	0.05	0.05	0.05	0.03	0.05

**Table 2:** The location of the holes.

No.	1	2	3	4
Angle (°)	0	30	60	90
Polar radius (mm)	5.5	6	6.5	7

**Table 3:** Imaging accuracy of LU-pSAFT.

	No.	1	2	3	4
Measured value	Central angle (°)	0.264	29.518	60.438	89.622
	Left limit (°)	−1.764	27.570	57.135	86.585
	Right limit (°)	2.131	31.810	63.438	92.773
	Polar radius (mm)	5.70	6.18	6.70	7.32
	Size (mm)	0.57	0.54	0.58	0.52
Absolute error	Central angle (°)	0.264	−0.481	0. 438	0.377
	Left limit (°)	0.687	0.916	0.687	0.343
	Right limit (°)	0.911	0.572	0.991	0.229
	Polar radius (mm)	−0.05	−0.07	−0.05	0.07
	Size (mm)	0.07	0.04	0.08	0.02

## Data Availability

The data presented in this study are available on request from the corresponding author.
